# Biochemical and kinetic characterization of laccase and manganese peroxidase from novel *Klebsiella pneumoniae* strains and their application in bioethanol production

**DOI:** 10.1039/c8ra01204k

**Published:** 2018-04-20

**Authors:** Nisha Gaur, Korrapati Narasimhulu, Pydisetty Y

**Affiliations:** Department of Biotechnology, National Institute of Technology Warangal 506004 Telangana India gaurnisha2007@gmail.com; Department of Chemical Engineering, National Institute of Technology Warangal 506004 Telangana India

## Abstract

Laccase (lac) and manganese peroxidase (MnP) enzymes from the novel *Klebsiella pneumoniae* isolates, grown on lignin basic media (LBM) were purified by 80% ammonium sulphate fractionation, dialysis and DEAE-sepharose column chromatography. The optimum temperatures for laccase production were 60 °C, 50 °C and 50 °C and for MnP production were 50 °C, 70 °C and 60 °C from NITW715076_2, NITW715076_1 and NITW715076 isolates, respectively. The optimal pH for production was found to be 5 for production of both the enzymes from all the isolates. 2.8–3.5 fold enzyme purification was achieved retaining around 60–70% of the initial activity. SDS-PAGE revealed the molecular mass of laccase and MnP to be 66 kDa and 48 kDa, respectively. The substrate ABTS and MnSO_4_ exhibited more specificity towards NITW715075_2 derived laccase and MnP (lac: *K*_m_ = 0.38 mM, *V*_max_ = 71.42 U ml^−1^; MnP: *K*_m_ = 0.17 mM, *V*_max_ = 106.38 U ml^−1^) compared to NITW715076_1 (lac: *K*_m_ = 3.97 mM, *V*_max_ = 148.8 U ml^−1^; MnP: *K*_m_ = 0.90 mM, *V*_max_ = 114.67 U ml^−1^) and NITW715076 (lac: *K*_m_ = 0.46 mM, *V*_max_ = 23.42 U ml^−1^; MnP: *K*_m_ = 0.19 mM, *V*_max_ = 108.10 U ml^−1^) derived. l-Cysteine and sodium azide imposed a strong inhibitory effect on the activities of both the enzymes. EDTA inhibited laccase and MnP activity at higher concentration. SDS strongly inhibited activity while for MnP it showed less inhibitory effect. The enzymes were employed for ethanol production from rice and wheat bran biomass which showed 39.29% improved production compared to control. After evaluating the applicability of these enzymes it can be suggested that the ligninolytic enzyme of *Klebsiella pneumoniae* isolates could be effectively employed in enhanced ethanol production and could be explored for other putative applications.

## Abstract 2

Upto 3 fold purified laccase and MnP from novel *Klebsiella* isolates, mediated ethanol production from rice and wheat bran substrates lead to almost 40% improvement in production profile.

## Graphical abstract

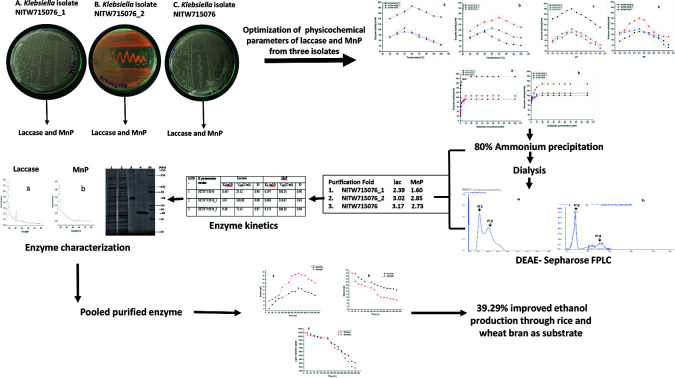

## 1. Introduction

Laccases (lac) (EC 1.10.3.2; benzenediol: oxygen oxidoreductases) belong to a group of copper containing glycoproteins with molecular weight between 60–100 kDa and are widely distributed among plants, fungi and bacteria.^1^ Manganese peroxidase (MnP) [EC 1.11.1.113; Mn(ii): H_2_O_2_ oxidoreductases] are also glycoproteins containing one 40–50 kDa protoporphyrin IX as a prosthetic group. In the past few decades both lac and MnPs have received considerable scientific attention for their versatile substrate specificity and ability to oxidise a wide range of phenols and polyphenols such as *ortho* and *para*-diphenols, methoxy-substituted phenols, phenolic acids and several other compounds. They are also capable of reducing molecular oxygen to water with one electron oxidation mechanism. The reactions catalysed by laccases and manganese peroxidases are very similar as both enzymes oxidize phenolic compounds and aromatic amines *via* one electron oxidation and form radicals. However, to the dissimilarities laccase is having lower oxidation potential than MnP.^1^ Both the enzymes play a vital role in detoxification and decolourization of pulp and paper mill effluent, textile effluent, ethanol production and bioremediation.^2^

Lac and MnP have been found to be widely distributed among plants and fungi but known laccase-like multi copper oxidases and peroxidases (MnPs) have been detected in the genome of many bacterial species. The presence of these ligninolytic enzyme in bacteria suggested that lac and MnP are also widespread in bacteria. However, from fungi the large number of laccase and MnP have been characterised and only few is from bacterial origin.^3^ Unlike ligninolytic fungi, ligninolytic bacteria can withstand high temperature and pH making them highly desirable candidate for environmental bioremediation, biofuel production, bio-pulping and textile industries and as biocatalysts for synthesis of value added products or structural modifications.^4,5^*Bacillus subtilis* SF, *B. halodurans*, *B. pumilus*, *B. subtilis* WPI, *Serratia marcescens*, *K. pneumoniae*, *Citrobacter* sp. and *C. freundii* FJ581026 are some of the reported microbial species for lac and MnP production.^6^ An increased number of characterised bacterial ligninolytic enzyme (lac and MnP) from various producers would allow taking advantage of its potential in development of novel application in bioprocess of green technology.

In this work production as well as purification of lac and MnP was done from *Klebsiella pneumoniae* strains NITW715076_1, NITW715076_2 and NITW715076 with the idea of realising the important potential application of these enzymes, particularly in ethanol production. Isolation characterization and identification of novel environmental strains capable of lac and MnP synthesis inspires further research focused on scaling up enzyme synthesis. The biochemical characterization and kinetic properties of enzymes were investigated. It is elucidated here that the same enzyme from different genera of same species possesses different biochemical and kinetic characteristics. Though laccase and MnP has many industrial applications such as detoxification of industrial effluent, enhanced ethanol production, uses as a medical diagnostic tool, fruit juice clarification *etc*.^5,7^ It is well known that the agricultural waste now a days used in the ethanol production contains lots of lignin. This lignin hinders in the ethanol production by producing phenolic moiety. So to reduce the lignin content and increasing the bioethanol production in this work, the purified enzymes were utilized for ethanol production.

## 2. Materials and methods

### 2.1. Bacterial strains and culture condition

The *Klebsiella pneumoniae* strains NITW715076 (accession no. KY435679), NITW715076_1 (accession no. MF086672) and NITW715076_2 (accession no. MF185143) used in this study were isolated from pulp and paper industry effluent collected from pulp and paper industrial area, Warangal, Telangana, India (18° 0′ 0′′ N and 79° 35′ 0′′ E). The collected effluent were stored at 4 °C and further used for microbial isolation. Lignin basic media (LBM) (g l^−1^): KH_2_PO_4_-1, yeast extract-0.01, ammonium tartrate diabasic-0.5, CuS0_4_·5H_2_0–0.001, MgSO_4_·7H_2_O-0.5, ferrous Sulphate-0.001, CaCl_2_·2H_2_O-0.01, MnSO_4_·H_2_O-0.001 and lignin-0.49. After autoclaving, 0.1% (w/v) glucose was added and media was used for isolation of potential laccase and MnP producing bacteria by serial dilution method. The cultures were maintained on lignin basic media at 35 °C with periodic transfer. The bacterial culture which showed the laccase and MnP activity were screened out by plate assay method (reference) and those showing enzyme activity (data not shown) were sent to Macrogen Inc. Korea for gene sequencing.

### 2.2. Enzyme production

For enzyme production lignin basic media was modified and synthetic lignin was replaced by mixing real waste water sample collected from pulp and paper industry wastewater for providing lignin exposure. Media was composed as follows (g l^−1^): KH_2_PO_4_-1, yeast extract-5, ammonium tartrate diabasic-0.5, CuS0_4_·5H_2_0–0.001, MgSO_4_·7H_2_O-0.5, ferrous sulphate-0.001, CaCl_2_·2H_2_O-0.01, and MnSO_4_·H_2_O-0.001. After autoclaving, 10 g l^−1^ sucrose was added to media.^8^ Prepared synthetic media was mixed with in 1 : 1 ratio. This modified media was used for optimization and production of laccase and MnP from *K. pneumoniae* strains. The broth was centrifuged at 8000 rpm for 20 min at 4 °C, and the supernatant was retained and stored at 4 °C for further purification and biochemical assays.

### 2.3. Enzyme activity assay

Laccase activity was measure by using method described by Atalla *et al.* (2013) based on the oxidation of the substrate 2,2′-azino-bis(3-ethylbenzothiazoline)-6-sulfonic acid (ABTS). The rate of oxidation of ABTS was determined spectrophotometrically at 420 nm. The reaction mixture contained 600 μl of 0.1 M citrate buffer (pH 5.0), 300 μl of 5 mM ABTS, 300 μl of crude enzyme and 1400 μl distilled water. After incubation of 2 min, the absorbance was measured. One unit of laccase activity was defined as the conversion of 1 mole of ABTS per minute in enzyme catalysed reaction.^9^

MnP activity was observed with oxidation of phenol red and the reaction mixture contained 500 μl of 0.1 M sodium acetate buffer (pH 5.6), 50 μl of 0.1% phenol red, 100 μl of 250 mM sodium lactate, 25 μl of 2 mM manganese sulphate, 100 μl of 0.5% bovine serum albumin (BSA) and 25 μl of 0.2 mM H_2_O_2_. MnP activity was measured spectrophotometrically by increase in Mn^3+^ malonate formation at 610 nm.^10^

### 2.4. Physicochemical properties of crude and purified enzyme

#### 2.4.1. Effect of different parameters on crude enzyme

##### 2.4.1.1. Effect of temperature

The effect of temperature on laccase and MnP activity was determined using ABTS and magnesium sulphate (MnSO_4_) as substrates respectively. The activities of crude enzymes were tested at different temperatures (30–100 °C) by standard enzyme assay at fixed pH 5.0 and substrate concentration (2 mM MnSO_4_ and 5 mM ABTS). Two different buffers 0.1 M sodium acetate and 0.1 M citrate buffer were used for MnP and laccase production respectively. After preparing the reaction mixture, different vials were incubated at different temperatures (30–100 °C) for 10 min and allowed to cool down to room temperature. Required mentioned amount of enzymes were afterwards taken in different test tubes for measuring its laccase and manganese peroxidase activity.

##### 2.4.1.2. Effect of pH

Optimum pH for crude enzymes was examined between the pH range of 2.5 to 8 with ABTS and magnesium sulphate as respective substrates at obtained optimal temperature and constant substrate concentration. The buffer systems used were sodium citrate for pH 2.0–5.0 and sodium acetate buffer for pH 5.7–8.0. 1 ml of the partial crude enzyme was incubated in the buffer solution of different pH range for 10 min at its optimum temperature. The laccase and MnP activity was checked once the samples attained room temperature.

##### 2.4.1.3. Effect of inhibitor concentration

The effect of inhibitors such as sodium dodecyl sulphate (SDS), sodium sulphite, l-cysteine, sodium azide and ethylene diamine tetra acetic acid (EDTA) on laccase and MnP activities was studied at varying concentration of 0.1 mM to 10 mM. A control was run parallelly in the absence of inhibitor. The partially purified laccase and MnP were incubated for 10 min at 50 °C and 60 °C respectively in 0.1 M citrate and sodium acetate buffer of pH 5.0 and 100 μl of varying concentration solution of each inhibitors was added.

##### 2.4.1.4. Effect of substrate concentration and kinetics

ABTS and MnSO_4_ were used as a substrate for laccase and MnP respectively in following concentration (mM) – 0.2, 0.5, 0.6, 0.8, 0.9, 1, 3, 5, 7, 10, 20, 30, 40, 50, 60, 70 and 100 at optimal temperature and pH. For both the laccase and MnP, enzyme blanks (using same substrate concentration without enzyme) were taken for blank measurements. In this experiment both the substrates of varying concentration were incubated with 1 ml partially purified enzyme and incubated for 10 min at optimum temperature and pH. After incubation, the laccase and MnP enzyme activities were checked once the vials attained room temperature. ABTS and MnSO_4_ were used to investigate the kinetic constant of laccase and MnP respectively. 0.1 M citrate buffer and sodium acetate buffer for laccase and MnP activity respectively were used to calculate *K*_m_ and *V*_max_ according to Lineweaver and Burk plot (plotted using origin pro 8) at optimum temperature and pH.^11^

#### 2.4.2. Protein quantification, purification and characterization

Bradford method was used to measure total protein (mg ml^−1^) and bovine serum albumin (BSA 1 mg ml^−1^) was used as a standard. Protein reagent was prepared according to Bradford method.^12^ In this method first protein reagent was prepared as followed: 100 mg Coomassie Brilliant Blue G-250 (Sigma) was dissolved in 50 ml of 95% ethanol. 100 ml of 85% phosphoric acid was added to this solution and the resulting solution was diluted to a final volume of 1 l. For preparing standard curve, BSA solution containing 10 to 100 μg protein were taken in the test tube. The volume was make up to 0.1 ml with 0.1 M citrate buffer. 5 ml of Bradford reagent was added to the test tube and mixed by vortexing. The absorbance was measured at 595 nm after 2 min and before 1 h against the reagent blank. The standard curve was used to determine the amount of protein in unknown sample and results were used to calculate the enzyme activity.

Crude enzymes (laccase and MnP) from NITW715076_2, NITW715076_1 and NITW715076 were precipitated by ammonium sulphate precipitation, where supernatant containing the crude enzymes were precipitated by 80% ammonium sulphate by vigorous shaking. The flasks were incubated at 4 °C for 24 h. The precipitated protein was collected thereafter by centrifugation at 10 000 rpm for 20 min. The sedimented protein was stored at 4 °C for dialysis.^13^

After ammonium sulphate precipitation, the concentration of both the enzyme and low molecular weight impurities increases. Therefore, to eliminate the impurities, dialysis was carried out *via* a dialysis membrane bag of 15 kDa cutoff. 500 ml 0.1 M citrate buffer (pH = 5.0) and 0.1 M sodium acetate buffer (pH = 5.6) was used for laccase and MnP respectively at 100 rpm with continuous stirring for 12 h at 4 °C. The dialysis was done in 3 steps to get more purified protein. In this process, 0.1 M citrate and sodium acetate buffer was used for laccase and MnP purification respectively. Buffers were changed in every 6 h repeating the same process again. The dialysis membrane containing crude protein was finally left in their respective buffers overnight.

Proteins were fractionated by gel filtration chromatography on DEAE-sepharose with fast performance liquid chromatography (FPLC) apparatus (AKTA prime Plus). For laccase purification, the column was equilibrated with 0.05 M Tris–HCl buffer (pH 7.2), and proteins were eluted at a linear 0.1 M citrate buffer (pH 5.0) gradient. For MnP, the equilibration was done by 0.1 M sodium acetate buffer (pH 5.6), and proteins were eluted at a linear 0.5 M NaCl gradient. The flow rate was 3 ml min^−1^ and fraction volume of 2 ml were collected at every 1 min interval.^1^ The fractions were assayed for determining enzyme activities and those having activities were pooled, concentrated by amicon (Merck, USA) and analysed by SDS-PAGE gel electrophoresis (major science).

#### 2.4.3. Molecular weight determination by SDS-PAGE

The molecular weight of laccase and MnP was analysed by 12% sodium dodecyl sulfate polyacrylamide gel electrophoresis (SDS-PAGE). The 12% SDS was prepared by resolving gel (separate the protein on the basis of their molecular weight) and stacking gel (concentrate all the protein in one band, so that they will start migrating in the running gel at the same time). The resolving gel (5 ml) was prepared as follow: 1.6 ml Milli Q water, 2.0 ml acrylamide (30 acrylamide : 0.2 bisacrylamide w/v), 1.3 ml 1.5 mM tris (pH 8.8), 50 μl 10% SDS, 50 μl ammonium persulfate and 5 μl tetramethylethylenediamine (TEMED). The stacking gel (3 ml) was prepared as follows: 2.1 ml Milli Q water, 500 μl acrylamide (30 acrylamide : 0.8 bisacrylamide w/v), 380 μl 1.5 mM tris (pH 6.8), 30 μl 10% SDS, 30 μl ammonium persulfate and 3 μl TEMED. Sample was prepared by mixing it with 2× loading dye. The sample was heated in boiling water for 10 min before loading into wells. The protein marker was also loaded along with samples and the sample was run at 100 V for 4 h. The gel was stained by coomassie brilliant blue (0.1% Coomassie Brilliant Blue R-250, 50% methanol and 10% glacial acetic acid) for 4 h and after that it was kept in de-staining solution (40% methanol and 10% glacial acetic acid) overnight. Unstained protein standards (10–250 kDa; New England Biolabs) were used to calculate the molecular weight of the enzymes. SDS-PAGE was performed using the discontinuous system (Laemmli, 1970).^14^

The laccase and MnP UV-absorbance spectrum was scanned within the range of 200 to 800 nm at room temperature using Jasco V-630 spectrophotometer.

### 2.5. Enzymes as inducer in bioethanol production

Wheat bran and rice bran-1 : 1 (RWB) were used as a substrate (carbon source) for bio-ethanol production. Acid hydrolysis was done by 2% H_2_SO_4_ followed by heating at 120 °C for 40 min.^15^ Lignin estimation in pre-treated and hydrolysed substrate was carried out as per described by Mishra *et al.*^16^ Briefly 50 ml sample was mixed with 1 ml of 10% CH_3_COOH and NaNO_2_. After 15 min, 2 ml of NH_4_OH was added and the absorbance was recorded spectrophotometrically at 430 nm after 5 min of incubation, taking blank measurement by replacing sample with distilled water while proceeding with same method. A test control was run parallelly in the absence of enzyme. The obtained absorbance values were converted into lignin content (ppm) by using following formula:
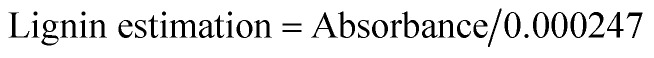


Total Reducing sugar test was done by 3,5-dinitrosalicylic acid (DNS) method.^15^ In brief, 100 μl sample was made upto 1 ml by diluting with distilled water and from 100 μl of sample was transferred into amber bottle. Following this, 900 μl of deionized water, 1 ml DNS reagent and 300 μl of 40% Rochelle salt was added to the amber bottle and kept in boiling water bath for 5 min. Absorption of the sample was read at 540 nm after sample was cooled. A test control was run parallelly in the absence of enzyme. Standard curve was prepared by using glucose as standard. Ethanol estimation was carried out by potassium dichromate oxidation method.^17^ Briefly, 1 drop of 0.1 M of silver nitrate was added to 5 ml of 0.25 M of potassium dichromate solution followed by addition of 5 ml of 6 M H_2_SO_4_. Afterwards 20 drops of unknown sample was added and incubated for 5 min following which 39 ml of distilled water was added and the absorbance was recorded at 560 nm. A test control was run parallelly in the absence of enzyme. Calibration curve was prepared using ethanol as standard.

Fermentation was carried out using hydrolysate obtained after pre-treatment. The hydrolysate was mixed with 3 ml of pure laccase and MnP and 0.1% w/v *Saccharomyces cerevisiae* for 384 h at 35 °C. Ethanol produced, lignin and reducing sugar were estimated regularly at every 12 h interval as per mentioned above.

## 3. Results and discussion

### 3.1. Effect of different parameters on crude protein

The optimized condition achieved for enzyme production was pH-6.5, temperature-35 °C, agitation speed-130 rpm, carbon source-sucrose (1%), nitrogen source-yeast extract (0.5%) and inoculum volume-4 ml/100 ml (data not shown). The following series of experiments were performed to investigate the effect of varying physicochemical parameters on activity of crude laccase and MnP.

#### 3.1.1. Temperature and pH profile

The activities of crude laccase and MnP from *Klebsiella pneumoniae* strains were examined at different temperatures with ABTS and MnSO_4_ as a substrate respectively and the enzymes were found to be active in a temperature range of 30–70 °C.

**Fig. 1:**
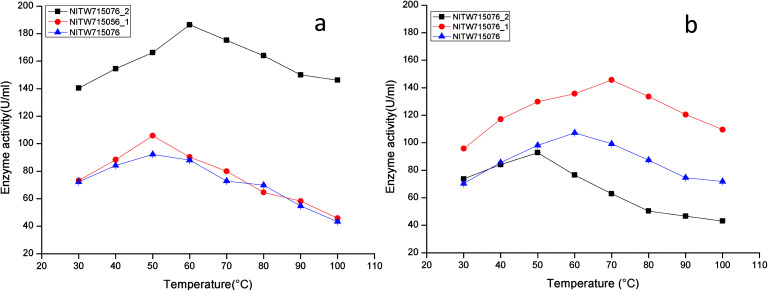
Temperature profile of (a) laccase and (b) MnP activity of *Klebsiella pneumoniae* strains NITW715076_2, NITW715076_1 and NITW715076.

The maximum laccase activity was found to be 186.41 ± 0.15 U ml^−1^ from strain NITW715076_2 at 60 °C after that the enzyme started losing its activity due to thermal instability. While in strains NITW715076_1 and NITW715076, the laccase activity was found to be 105.83 ± 0.36 U ml^−1^ and 92.28 ± 0.4 U ml^−1^ respectively at 50 °C (Fig. 1a). Similar optimum temperature was found for laccase from *Trametes* sp.^18^ Rezaei *et al.* reported maximum activity of laccase from *Aquisalibacillus elongates* at 40 °C.^19^ In another finding, *Bacillus subtilis* MTCC 2414 showed the maximum activity at 40 °C.^2^

The maximum MnP activity for NITW715076_2 was observed 92.77 ± 0.12 U ml^−1^ at 50 °C. For strains NITW715076_1 and NITW715076 the MnP activity was found to be 145.65 ± 0.46 U ml^−1^ at 70 °C and 107.24 ± 0.41U ml^−1^ at 60 °C respectively (Fig. 1b). This enzyme has not been much explored in bacteria, but few decade ago Oliveira *et al.*, studied this enzyme from *Bacillus pumilus* and *Paenibacillus* sp. and found 25 and 35 °C as optimum temperature respectively for MnP activity, contrasting to our observation.^20^ Fungal origin of this enzyme has also been reported which showed the similar (high) optimum temperature as found in this study. In a recent investigation, researchers showed the characterization of a novel MnP from *Echinodontium taxodii* 2538 and they found 55 °C to be optimum for maximum MnP activity.^21^ In another study, MnP from *Ganoderma lucidum* IBL-05 showed the maximum activity at 60 °C. The reported high optimum temperatures for maximum MnP activity are in alignment to the findings of the current study.^22^

The observations suggest that the bacterial ligninolytic enzymes can also show high thermal stability as fungal enzymes NITW715076_1 and NITW715076 lost 56.6% and 53% of its laccase activity after 30 min of incubation at 50 °C respectively. However, NITW715076_2 lost relatively lesser *i.e.* 21.5% laccase activity after 30 min of incubation at 60 °C. From the literature review, it is confirmed that the optimal temperature of laccase and MnP differs greatly from one strain to another. The reason behind this may be different environmental condition and stress mediated microbial enzyme production and utilization of different nutrients for its metabolic activity and enzyme production. The results of this study confirmed that the laccase and MnP from *K. pneumoniae* isolates are highly thermostable and can withstand temperature upto 70 °C. The high thermal stability of these enzymes makes them desirable candidates for various industrial applications.

**Fig. 2:**
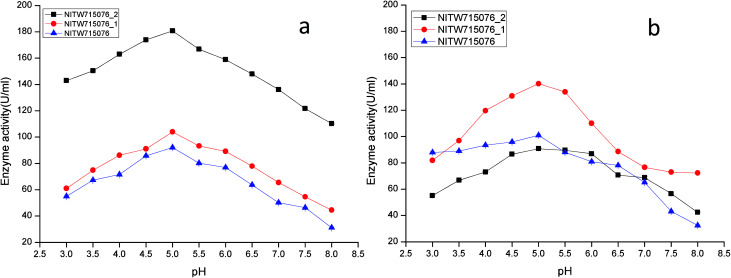
pH profile of (a) laccase and (b) MnP activity *Klebsiella pneumoniae* strains NITW715076_2, NITW715076_1 and NITW715076.

The effect of pH on the enzyme activity was measured over a range of 3.0–8 taking same respective substrates ABTS and MnSO_4_. pH 5 was found to be suitable for maximum activity of crude laccase from bacterial strains NITW715076_2 (180.70 ± 0.5 U ml^−1^), NITW715076_1 (104.02 ± 0.5 U ml^−1^) and NITW715076 (92.06 ± 0.4 U ml^−1^) (Fig. 2a). The optimum pH for enzyme activity varies with the type of substrate used due to the difference in the redox potential of the type 1 copper of laccase and the substrate. In case of alkaline range, the optimum pH of phenolic substrates varies with redox potential difference between the phenol and the T1 copper of laccase and hence increases accordingly.^23,24^ With ABTS, pH 5 was found optimum for laccase of all three *K. pneumoniae* isolates which can be explained by non-phenol nature of ABTS. From these results it can be inferred that the laccase from all bacterial strains is slightly acidic and can be used in industries working with acidic pH conditions. Laccase from *Bacillus tequilensis* SN4 also showed optimum enzyme activity with ABTS at pH 5.5.^25^ Siroosi *et al.* did the similar work with *Bacillus* sp. strain WT and they also observed higher laccase activity towards ABTS at acidic pH between 4.0–5.0.^26^ These results are in alignment to current observations and hence indicates that with ABTS substrate the bacterial laccases usually show higher activity in acidic pH.

MnP activity was investigated at pH range of 3.0–8 with MnSO_4_ substrate. Maximal enzyme activities were observed at pH 5.0 notably 90.78 ± 0.5 U ml^−1^, 140.22.16 ± 0.5 U ml^−1^, and 100.99 ± 0.49 U ml^−1^ from NITW715076_2, NITW715076_1 and NITW715076 strains respectively (Fig. 2b). In all bacterial strains MnP lost its activity ∼45–50% over pH 5.0. Oliveira and co-workers worked on *Bacillus pumilus* and *Paenibacillus* sp. MnP and reported its highest activity at pH 8.0 and 9.0 respectively contrasting to our observations. As already mentioned, this enzyme is less explored in bacteria in contrast to fungi which had the similar optimal working conditions what we have found in our work. MnP from *Pleurotus pulmonarius* was active over a large range of pH 4.0–6.0 for MnSO_4_.^27^ Similarly, MnP from *Irpex lacteus* F17 and was found to have maximum enzyme activity over a broad pH range of 4–7.^28^ Both laccase and MnP enzymes showed maximum activity in acidic range over alkaline pH range.

#### 3.1.2. Effect of substrate concentration and kinetic studies

**Fig. 3:**
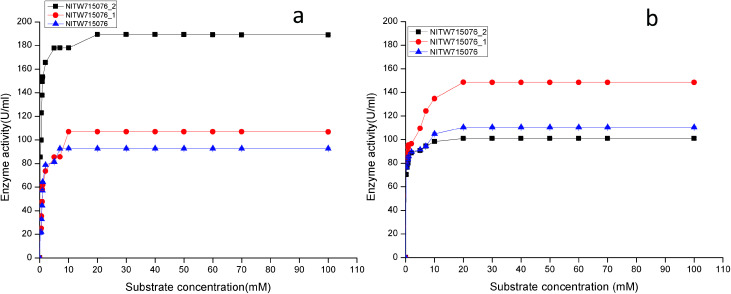
Effect of substrate concentration on (a) laccase and (b) MnP activity *Klebsiella pneumoniae* strains NITW715076_2, NITW715076_1 and NITW715076.

The effect of different substrates on laccase (pyrogallol, tannic acid, syringaldazine, ABTS, guaiacol *etc.*) and MnP (MnSO_4_, H_2_O_2_, guaiacol, ABTS *etc.*) production has been well studied previously and ABTS and MnSO_4_ are found to be effective substrates.^19,21^ Therefore in this study these two substrates only, were selected and their different concentrations were evaluated to determine the one exhibiting maximum enzyme activity. Results in Fig. 3a and b shows the linear increase in laccase and MnP activities on increasing the ABTS and MnSO_4_ concentration respectively. Higher substrate concentrations resulted in stable enzyme activity. In case of laccase, enzymes from strains NITW715076_2, NITW715076_1 and NITW715076 showed maximum activity at 20 mM, 10 mM and 7 mM respectively. The effect of MnSO_4_ concentration on MnP enzyme activity indicated that the activity increased linearly upon increasing the MnSO_4_ concentration up to 20 mM for all three isolates, after that enzyme activity became constant (Fig. 3b).

Using ABTS and MnSO_4_ as substrate, the *K*_m_ and *V*_max_ values of purified laccase and MnP from *K. pneumoniae* strains were determined from Lineweaver and Burk plots. The *K*_m_ and *V*_max_ values of both the enzymes are presented in Table 1. All bacterial strains were able to oxidise ABTS, and it could be concluded that it is a true laccase. The *K*_m_ of NITW715076_2 showed the highest binding affinity towards the ABTS (having two hydroxyl groups that may be beneficial for enzyme action) and MnSO_4_ with lowest *K*_m_ and maximum *V*_max_ as compared to NITW715076_1 and NITW715076. Similar results have been observed for *Streptomyces psammoticus*,^29^*Bacillus* sp. strain WT,^26^*Echinodontium taxodii* 2538.^21^ So from these results it can be inferred that the similarity is due to the same substrate and nearly similar pH, temperature and culture condition used.

**Table 1:** Comparison of *K*_m_ and *V*_max_ of laccase and MnP from various *Klebsiella pneumoniae* strains

S no.	*K. pneumoniae* strains	Laccase			MnP		
		*K*_m_ (mM)	*V*_max_ (U ml^−1^)	*R* ^2^	*K*_m_ (mM)	*V*_max_(U ml^−1^)	*R* ^2^
1	NITW715076	0.467	23.42	0.96	0.197	108.10	0.90
2	NITW715076_1	3.97	148.80	0.98	0.904	114.67	0.93
3	NITW715076_2	0.38	71.42	0.97	0.173	106.38	0.94

#### 3.1.3. Effect of inhibitors

The effect of various chemicals and compounds (sodium sulphite, l-cysteine, EDTA, SDS and sodium azide) on laccase and MnP activity was investigated (Table 2). Among inhibitors, l-cysteine showed the highest inhibitory effect on enzyme activity as it is a reducing agent and reduced the oxidized substrate of laccase. While NaN_3_ almost had similar inhibitory effect even at lower concentration as azides binds to the copper atom of laccase. Another well-known metal chelating agent EDTA, fully inhibited the laccase activity at higher concentration probably by chelating copper atom of laccase.^25^ Furthermore, SDS strongly inhibited the laccase activity even at lower concentration by denaturizing the laccase. Laccase from *Bacillus* sp. strain WT were inhibited by SDS at 1 mM concentration and showed relative activity 61.4%.^26^ There are controversial reports about the EDTA and sodium azide in which EDTA had no inhibitory effect on enzyme even at higher concentration (25 mM) while sodium azide has exerted little inhibition on laccase even at higher concentration (10 mM).^30^

**Table 2:** Effect of various concentration of inhibitors on laccase and MnP activity from *Klebsiella pneumoniae* strains

S. no.	Inhibitor	Concentration(mM)	Inhibition (%)	
			Laccase	MnP
1.	Control	0	0	0
2.	Sodium sulphite	0.1	5	11.22
		2	20	30.68
		5	83.72	77.33
		10	97.5	84.21
3.	l-Cysteine	0.1	79	65.21
		2	95.29	84.83
		5	99	97.01
		10	100	100
4.	EDTA	0.1	0	1.61
		2	15.75	45.45
		5	40.36	73.22
		10	90.24	100
5.	SDS	0.1	25.7	18.31
		2	48.6	27.02
		5	95.2	32.11
		10	100	52.4
6.	Sodium azide	0.1	38.3	44.06
		2	67.31	78.91
		5	86.9	89.33
		10	90.2	99

Sodium azide and l-cysteine had strong inhibitory effect on MnP activity even at lower concentrations. Sodium azide can cleave disulfide bonds present in active site of MnP structure (has five disulphide-bridging elements). The enzyme activity disappeared completely with increase in EDTA concentration (10 mM). Since EDTA is metal chelating agent it has ability to complex with inorganic complex or metal ions, because of which it might have chelated Mn^3+^ and competed with the ion rather than inhibit the enzyme itself. SDS degrades the protein structure (tertiary or quaternary) by destroying the hydrophobic effect. The addition of SDS had less inhibitory effect on MnP activity even at 10 mM concentration suggesting that MnP from *K. pneumoniae* isolates were tolerance to SDS.^21,31,32^ Both enzyme were partially inhibited by sodium sulphite at lower concentration, which at higher concentration completely inhibited the enzyme, evident from little enzyme activity.

### 3.2. Enzyme purification and characterization

#### 3.2.1. Purification of laccase and MnP

The culture supernatant containing crude enzyme, of *K. pneumoniae* strains were subjected to further purification. Enzymes were partially purified by 80% ammonium sulphate precipitation. After dialysis, the specific activity, and purification fold of laccase and MnP were increased in all strains. The specific activity of laccase from isolate NITW715076_2 increased from 38.85 to 123.39 U mg^−1^ and of MnP increased from 29.40 to 84.04 U mg^−1^. The final yield (yield% = divide total enzyme activity by initial total activity multiply by 100) of pure enzyme was 48.6% and 68.68% and purity was 3.02 and 2.85 fold higher in laccase and MnP respectively as compared to other bacterial strains of *K. pneumoniae* (Table 3). In isolate NITW715076_1, the specific activity of laccase increased from 91.12 U mg^−1^ to 217.89 U mg^−1^ and in MnP increased from 63.61 to 101.85. The purification in laccase and MnP was 2.39 and 1.60 fold which corresponded to final yield of 61.25% and 55.62% respectively (Table 4).

**Table 3:** Purification of laccase and MnP from the culture filtrate NITW715076_2

Purification steps	Enzyme activity (U ml^−1^)		Total protein (mg ml^−1^)		Specific activity (U mg^−1^)		Yield (%)		Purification fold	
	Lac	MnP	Lac	MnP	Lac	MnP	Lac	MnP	Lac	MnP
Crude filtrate	190.38	102.91	4.90	3.5	38.85	29.40	100	100	1	1
Dialysis (15 kDa cut off)	150.61	88.36	1.72	1.2	87.56	72.42	89.6	85.86	2.14	2.46
DEAE-sepharose	92.54	70.62	0.75	0.84	123.39	84.08	48.6	68.63	3.02	2.85

**Table 4:** Purification of laccase and MnP from the culture filtrate NITW715076_1

Purification steps	Enzyme activity (U ml^−1^)		Total protein (mg ml^−1^)		Specific activity (U mg^−1^)		Yield (%)		Purification fold	
	Lac	MnP	Lac	MnP	Lac	MnP	Lac	MnP	Lac	MnP
Crude filtrate	110.26	150.13	1.21	2.36	91.12	63.61	100	100	1	1
Dialysis (15 kDa cut off)	96.38	102.42	0.52	1.2	185.36	85.35	87.4	68.2	2.03	1.32
DEAE-sepharose	67.54	83.52	0.31	0.82	217.89	101.85	61.25	55.62	2.39	1.60

Table 5 shows the recovery and yield of laccase and MnP from culture filtrate NITW715076. The specific activity of laccase and MnP was 194.18 and 241.18 U mg^−1^ while the purification was 3.17 and 2.73 fold, which corresponded to final yield of 61.58 and 68.99 respectively. The obtained results differ with those obtained from a few studies on laccase and MnP purification by DEAE-sepharose column chromatography.^26,29^ The two step purification performed in this study for laccase and MnP enabled us to achieve a purification of about 2.5 to 3.5 folds and a final yield of 60–70% with respect to enzyme activity.

**Table 5:** Purification of laccase and MnP from the culture filtrate NITW715076

Purification steps	Enzyme activity (U ml^−1^)		Total protein (mg ml^−1^)		Specific activity (U mg^−1^)		Yield (%)		Purification fold	
	Lac	MnP	Lac	MnP	Lac	MnP	Lac	MnP	Lac	MnP
Crude filtrate	97.75	115.35	1.6	1.31	61.09	88.05	100	100	1	1
Dialysis (15 kDa cut off)	84.38	99.36	0.9	0.75	93.75	132.4	86.32	86.1	1.53	1.5
DEAE-sepharose	60.19	79.59	0.31	0.33	194.18	241.18	61.58	68.99	3.17	2.73

**Fig. 4:**
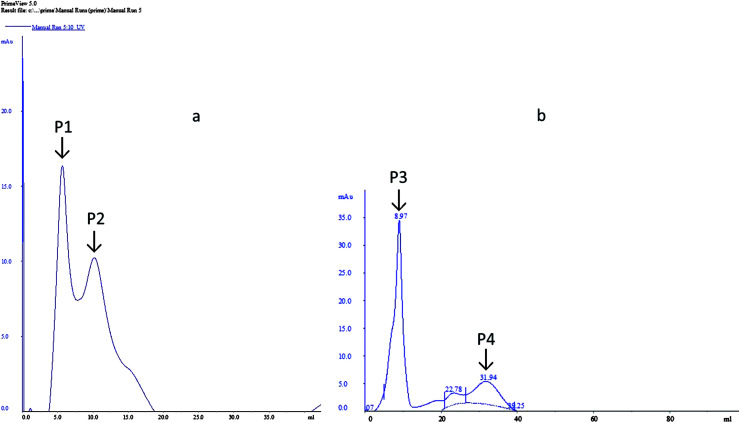
Purification of extracellular enzyme from *Klebsiella pneumoniae* strains. (a) Laccase (b) MnP.

The dialyzed samples were applied to DEAE-sepharose column chromatography (Fig. 4). Peak P1, which is adsorbed on DEAE-sepharose and eluted with 0.05 M Tris–HCL, showed the highest laccase activity (92.54 U ml^−1^). While for peak 2 the laccase activity (76.39 U ml^−1^) was comparatively less. However, for MnP two peaks were obtained, a large absorbed peak P3 showed the highest MnP activity (83.52 U ml^−1^) and a small peak P4 was devoid of MnP activity.

#### 3.2.2. Enzyme characterization

##### 3.2.2.1 SDS-PAGE

**Fig. 5:**
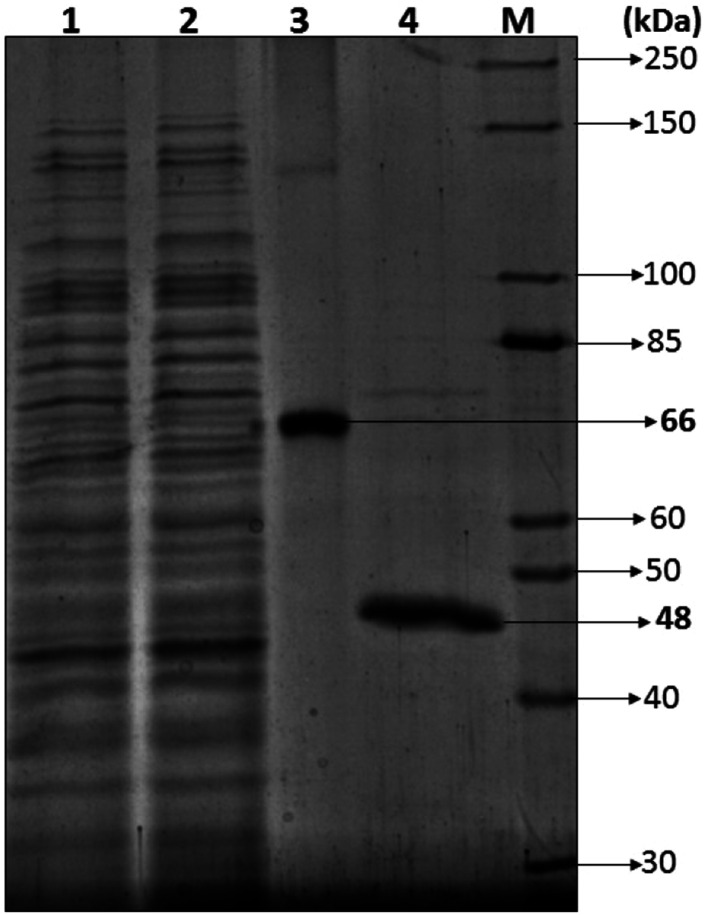
Estimation of the molecular mass of *Klebsiella pneumoniae* laccase and MnP *via* SDS-PAGE (12%): (1) crude sample, (2) sample after dialysis, (3) purified laccase, (4) purified MnP, M. Unstained protein ladder (10–250 kDa).

Most of the bacterial and fungal laccases and MnP reported till date are either intracellular or spore bound making their industrial application unfeasible.^33–36^ Laccase and MnP from *K. pneumoniae* strains are extracellular and thermo-stable making them attractive candidates for industrial application. In order to determine the molecular weight of laccase and MnP protein, produced by *K. pneumoniae* strains, crude laccase and MnP were subjected to SDS-PAGE. The extracellular laccase and MnP enzyme were purified to homogeneity, as were evident from the appearance of single band on SDS-PAGE. All strains produced laccase and MnP enzyme of molecular weight of 66 kDa and 48 kDa respectively (Fig. 5).

The molecular weight of all reported bacterial^37^ and fungal^38^ laccases have also been found to be in the range of 50–100 kDa. The molecular weight of *Klebsiella pneumoniae* isolates MnP is 48 kDa which is very high as compared to other bacterial MnPs^20^ but fungal MnPs^32,39^ generally lie in this range only *i.e.* 30–50 kDa. This difference in the molecular weight makes *K. pneumoniae* MnP an interesting protein for its structural and functional relationship. The anonymity could be further explored to understand it better.

##### 3.2.2.2 UV-spectral analysis

**Fig. 6:**
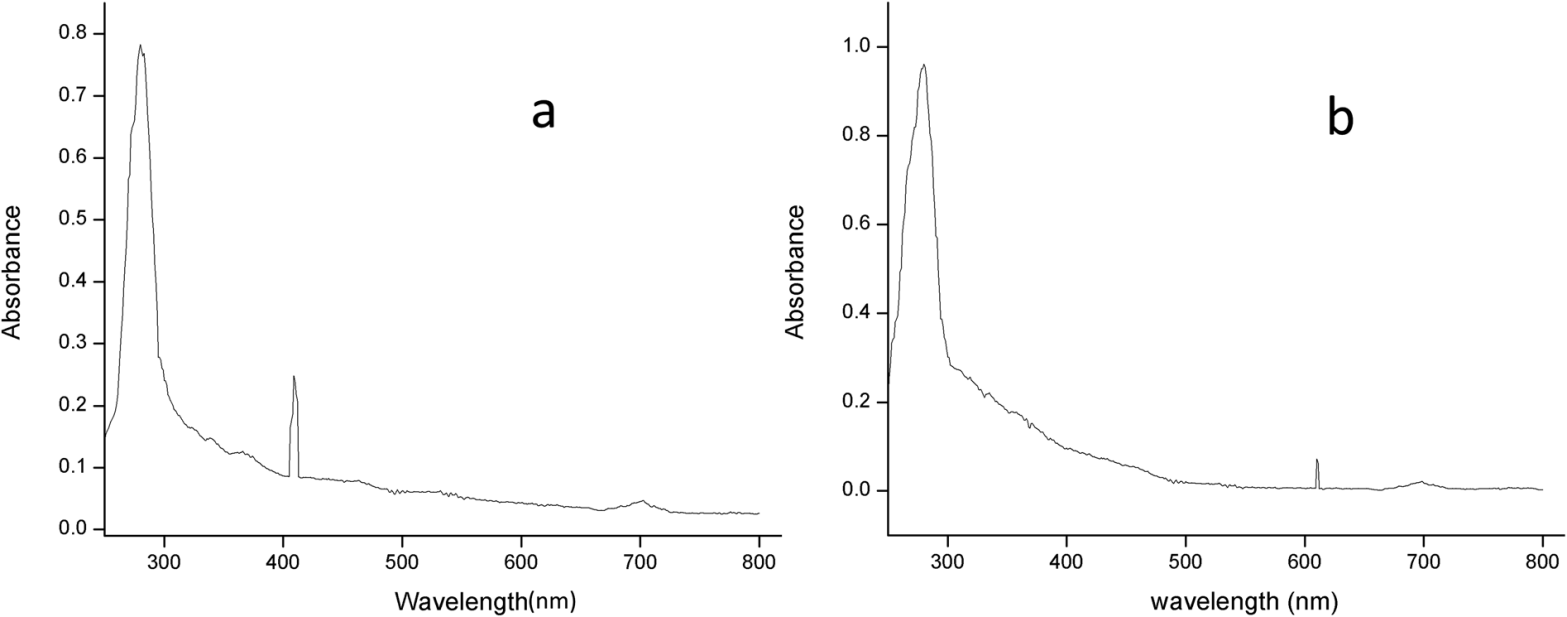
The absorption spectrum of (a) laccase and (b) MnP from *Klebsiella pneumoniae* strains at room temperature in 0.1 M citrate buffer (pH 5.0) and sodium acetate buffer (pH 5.6).

The absorption spectrum of the purified laccase showed maxima at about 280 and 610 nm (Fig. 6a). The enzyme was a typical blue laccase as it showed an intense band around 610 nm, corresponding to the type-1 copper site which is responsible for the deep blue colour of the enzyme. The slight peak near 300 nm corresponding to the presence of the type-3 copper site at typical blue laccase.^18^Fig. 6b showed the maxima at 280 and 409 nm for purified MnP, these spectral characteristics are very similar to those of MnP from *Irpex lacteus* and *Phlebia radiate*, suggesting that the enzyme is a heme protein with iron protoporphyrin IX as a prosthetic group.^40^

### 3.3. Application of enzyme in bioethanol production

There has been increasing interest from last few decades in utilizing agro-wastes as inexpensive substrates for economical production of variety of value added products. In present work also we utilized rice and wheat bran as inexpensive substrate for ethanol production to evaluate the practical applicability of the produced enzymes laccase and MnP.^41^ Laccase and MnP from *K. pneumoniae* strains were added in the inexpensive raw materials rice and wheat bran hydrolysate to improve the production of ethanol, these enzymes can play crucial role in increasing the ethanol production by degrading phenolic compounds present in the lignocellulose hydrolysate. Removal of lignin content from RWB biomass by mild alkali treatment was essential in order to decrease lignin content. Initially, RWB was alkali pre-treated and lignin content obtained after this process was 11 030 ± 0.07 ppm. To increase saccharification efficiency, after alkali treatment, RWB was acid hydrolysed. Reducing sugar and lignin content post hydrolysis were 36.133 ± 0.05 mg ml^−1^ and 10 990.51 ± 0.04 ppm. In some previous investigations also pre-treatment approach resulted in significant lignin reduction making the celluloses more accessible for saccharification.^15,42,43^

**Fig. 7:**
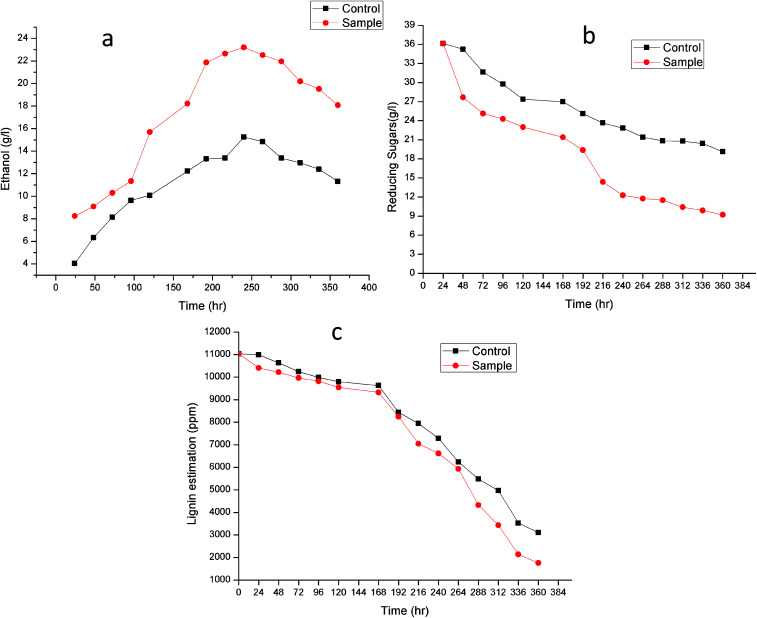
Time course for (a) ethanol production, (b) reducing sugar consumption and (c) lignin degradation.

Fig. 7 shows the time course for (a) Ethanol production (b) reducing sugar consumption and (c) lignin degradation. Alkali treatment generates irregular pores and disrupt the lignin-carbohydrate complex so that hydrolytic enzyme can work on carbohydrate breakdown into simpler one. This lignin disruption can be further enhances by use of laccase and MnP treatment to obtain high delignification efficiency, increase the porosity and the available surface area as well as decrease the non-specific adsorption of hydrolytic enzymes. In addition, laccase can work as a detoxification agent to improve the ferment-ability of pre-treated lignocellulosic. Phenols not only affect the biological membranes but also affect the growth rate, inhibit and deactivate the hydrolytic enzymes.^44–46^

Lignin estimation (Fig. 7c) was also done at regular interval of time, at 24 h the lignin was 10 990.51 ± 0.07 ppm and 10 406.33 ± 0.09 in control (without enzyme) and sample respectively. It started decreasing because of presence of ligninolytic enzyme. At 360 h the lignin content was 3103.42 ± 0.04 ppm and 1760.23 ± 0.05 ppm in control and sample respectively. Recently Ire *et al.* produced bioethanol from steam-exploded bagasse, initially the lignin content was 19.2 ± 1.2% (w/w) which was reduced to 4.2 ± 0.44% (w/w) by acid treatment. But in this present study the lignin degradation was done by real enzymes produced by bacteria to reduce the contamination already present in water. They worked on cellulose and xylanase producing bacteria for enhanced ethanol production.^43^ In another study, delignification from different agricultural waste such as corn cobs (48.05 ± 2.4%), corn stover (49.52 ± 1.5%), rice straw (43.45 ± 1.3%), banana stalk (39.15 ± 0.9%) and sugarcane bagasse (56.9 ± 2.1%) by ligninolytic enzyme and found maximum ethanol production (23.36 ± 2.3 g l^−1^) from sugarcane bagasse.^47^

Laccase catalyse the oxidation of phenols by generating unstable phenoxy radicals and these radicals interact with each other which leads to the polymerization into aromatic compounds (having lower inhibitory capacity). Due to structural characteristic of phenols impose difficulties in its complete degradation and elimination, laccase converts certain compounds (syryngaldehyde, cinnamic acids *etc.*) or oxidize other phenolic compounds into simpler one (vanillin).^48–50^

Similarly, MnP generates Mn^3+^ (act as a diffusible charge-transfer mediators) which can oxidize a large amount of phenolic substrates such as simple phenols, amines, dyes as well as phenolic lignin model compounds. In contrast to laccase, MnP is not capable of oxidizing the more recalcitrant non-phenolic compounds. However it has been reported that if a bacteria is producing laccase and MnP but not LiP then MnP can cleave non-phenolic lignin substrates *via* the action of small mediators such as thiol and lipid radicals.^51,52^

The increase in microbial growth in present work was found to be inversely proportional to glucose concentration in fermentation medium. At 24 h the glucose concentration was 36.133 ± 0.05 g l^−1^ however as the ethanol starts producing, glucose concentration in control and sample started decreasing and it reached to 19.12 ± 0.02 g l^−1^ and 9.203 ± 0.03 g l^−1^ respectively at 360 h. It was logically due to utilization of the glucose (nutrient source) in the fermentation medium by *Saccharomyces cerevisiae*. During fermentation process the pH of the medium changed due to production of ethanol as well as other organic acids.^53^ The results showed gradual increase in ethanol up to 240 h in control (without enzyme) (15.25 ± 0.03 g l^−1^) and sample (23.21 ± 0.06 g l^−1^) after that it declined in both the cases. From steam-exploded bagasse Ire *et al.* attained 19.08 g l^−1^ ethanol fermented by co-culture of *B. cereus* and *B. thuringiensis*.^43^ In case of banana pseudo stem Ingale *et al.* achieved 17.1 g l^−1^ ethanol.^54^ Recently, Ezebuiro *et al.* reported ethanol yield of 18.40 g l^−1^ and 17.80 g l^−1^ by *Bacillus cereus* using sugarcane bagasse and cassava peels respectively.^55^ It is interesting to note that ethanol yield in the present work is comparatively high as compared to previous reports. Most of the bacterial and fungal laccases and MnP reported till date are either intracellular or spore bound making their industrial application unfeasible.^33–36^ Laccase and MnP from *K. pneumoniae* strains reported in this study are extracellular and thermo-stable making them attractive candidates for industrial application. Moreover the present work also practically elucidate their application in enhanced ethanol production (higher than any of the previous reports) suggesting them potent enzymes for ethanol production as well as other industrial applications.

## 4. Conclusion

Laccase and MnP secreted from *Klebsiella pneumoniae* strains were purified and characterized. Both enzymes exhibited high catalytic efficiency towards ligninolytic substrates than any other bacterial laccase and MnP. In addition, the purification of laccase and MnP from the culture filtrate of all strains showed 2.5 to 3.5 fold and final yield of 60–70% as well as molecular weight of 66 kDa and 48 kDa respectively. Moreover, laccase and MnP secreted from *Klebsiella pneumoniae* strains are thermally stable and showed maximum activity in acidic range of pH over alkaline pH range. Strains NITW715076_2, NITW715076_1 and NITW715076 exhibited maximum laccase activity at 20 mM, 10 mM and 7 mM respectively while for MnP it was 20 mM after that it became constant.

Laccase and MnP from NITW715076_2 showed the higher substrate affinity and catalytic efficiency as its *K*_m_ is low as compare to other stains. The characterization of laccase and MnP with various pH, temperature, substrate concentration, and different inhibitors suggested that these enzymes could be potentially useful for other biotechnological applications. Our investigations revealed a 39.29% increase in ethanol as compared to control utilizing the laccase and MnP from *Klebsiella pneumoniae* isolates suggesting its potent applicability. Therefore, these enzymes could be used in ethanol production and they can also find potential applicability in decolourization and detoxification of paper mill effluent.

## Conflicts of interest

All authors mutually declare that they do not have any conflict of interest.

## Supplementary Material
